# Split-Ring Resonator Sensor Penetration Depth Assessment Using In Vivo Microwave Reflectivity and Ultrasound Measurements for Lower Extremity Trauma Rehabilitation

**DOI:** 10.3390/s18020636

**Published:** 2018-02-21

**Authors:** Syaiful Redzwan Mohd Shah, Jacob Velander, Parul Mathur, Mauricio D. Perez, Noor Badariah Asan, Dhanesh G. Kurup, Taco J. Blokhuis, Robin Augustine

**Affiliations:** 1 Microwaves in Medical Engineering Group, Solid State Electronics, Department of Engineering Sciences, Angstrom Laboratory, Uppsala University, Box 534, SE 75121 Uppsala, Sweden; Jacob.Velander@angstrom.uu.se (J.V.); mauriciodavidperez@gmail.com (M.D.P.); noorbadariah.asan@angstrom.uu.se (N.B.A.); 2 Department of Electronics and Communication, Amrita School of Engineering, Amrita Vishwa Vidyapeetham, Bengaluru , 560035, India; p_mathur@blr.amrita.edu (P.M.); dg_kurup@blr.amrita.edu (D.G.K.); 3 Department of surgery, Maastricht University Medical Center, PO Box 5800 6202 AZ Maastricht, The Netherlands; taco.blokhuis@mumc.nl

**Keywords:** Microwave measurement, ultrasound measurement, split-ring resonator, penetration depth, human lower extremity, sensor, model optimization, multilayered material, electric field distribution

## Abstract

In recent research, microwave sensors have been used to follow up the recovery of lower extremity trauma patients. This is done mainly by monitoring the changes of dielectric properties of lower limb tissues such as skin, fat, muscle, and bone. As part of the characterization of the microwave sensor, it is crucial to assess the signal penetration in in vivo tissues. This work presents a new approach for investigating the penetration depth of planar microwave sensors based on the Split-Ring Resonator in the in vivo context of the femoral area. This approach is based on the optimization of a 3D simulation model using the platform of CST Microwave Studio and consisting of a sensor of the considered type and a multilayered material representing the femoral area. The geometry of the layered material is built based on information from ultrasound images and includes mainly the thicknesses of skin, fat, and muscle tissues. The optimization target is the measured S_11_ parameters at the sensor connector and the fitting parameters are the permittivity of each layer of the material. Four positions in the femoral area (two at distal and two at thigh) in four volunteers are considered for the in vivo study. The penetration depths are finally calculated with the help of the electric field distribution in simulations of the optimized model for each one of the 16 considered positions. The numerical results show that positions at the thigh contribute the highest penetration values of up to 17.5 mm. This finding has a high significance in planning in vitro penetration depth measurements and other tests that are going to be performed in the future.

## 1. Introduction

Penetration depth performance of sensors has been studied for many decades for multipurpose uses [1,2,3,4]. It could decisively affect how electromagnetic (EM) waves are absorbed, transmitted, and reflected in materials, especially in biological tissues. These penetration depth performances are used in dosimetry studies, including microwave technology, and they have brought about many new exciting therapeutic and diagnostic solutions [5,6]. Recent studies show that the association of the microwave technique with biological applications is growing rapidly [7,8,9,10,11,12,13]. 

For several reasons, hip fractures could be a valuable clinical application of this new technique. Hip fractures are one of the most common health issues among the elderly population nowadays as they cause significant morbidity and are often associated with increased mortality. Moreover, hip fractures lead to decreased mobility, mostly due to loss of muscle mass. Rehabilitation after a hip fracture is guided by clinical and radiological monitoring. For radiological monitoring, the gold standard is the use of X-ray, dual-energy X-ray absorptiometry (DEXA), and computed tomography (CT) images [14,15,16]. However, these techniques all have technical limitations and only provide some insight into the condition of calcified tissues. Clinical monitoring is limited as well, as it is guided by calculating mobility scores and observing gait patterns. Either monitoring method is not suitable to estimate the rehabilitation process, nor to determine the phase of rehabilitation for an individual at a certain time point. This leads to uncertainty for patients and care providers, but also to inefficiency in directing care to the appropriate patient who needs it most. Given the shortcomings of current monitoring techniques, the same rehabilitation regimen is imposed on all patients, instead of providing individualized care for those who need it and only observation and limited guidance for those who do well [17,18]. 

Therefore, there is an important clinical need to develop an effective, safe, portable, and rapid method for assessing the different healing stages of lower extremity injuries, such as hip fractures. A better understanding of the effective variations of the tissues affected during the rehabilitation of lower extremity fractures could lead to promising devices. Specifically, monitoring muscle tissue reliably may change clinical practice in the future. Our method is based on this observation as the key to answering that social need. Therefore, to understand those tissue variations from the clinical perspective, a comprehensive investigation is necessary and important. These effective variations could be correlated to the different healing stages, as can be seen in [19,20].

In a recent publication by Perez et al. [21], the authors demonstrated clinical measurement using Split-Ring Resonator (SRR) sensors operating at microwave frequency. The sensor has a large influence on the complementary dimension of the ring resonator compared to the T-coupling and ring resonator with the multilayer substrate. However, it gives minimal insight into the resolution with respect to the resonance frequency shift.

This paper presents the measurement and numerical analysis of an SRR sensor operating at the industrial, scientific, and medical radio (ISM) band (2.4–2.5 GHz) for studying the penetration depth in particular layers of multilayered biological tissue in terms of resonance frequency and permittivity variations. We utilize the same measurement configuration but with a range of different points on the femoral area to illustrate that the penetration depth is closely related to the variation of the biological tissue.

This paper is organized as follows. Section 2 describes the outline of material and methods of the sensor selectivity, clinical measurement approach, and numerical modelling procedures. Meanwhile, Section 3 explains in detail the results and discussion on the feature dependence of resonance frequency on position, permittivity analysis, and penetration depth and penetration analysis; this is followed by conclusions in Section 4.

## 2. Materials and Methods 

### 2.1. Sensor Selection

In this study we used two designs of sensor—Split-Ring Resonator (SRR) and Capacitive Split-Ring Resonator (CSRR)—based on a previous study by Perez et al. [21]. These sensors were used to observe the penetration depth from different parameters such as resonance frequency *f_r_*, effective permittivity *ε_eff_*, and tissue thickness *t_s_*. Furthermore, the signal that penetrated through different tissue layers was investigated in terms of its electric field (E-field) to analyze the signal penetration depth. 

**Figure 1:**
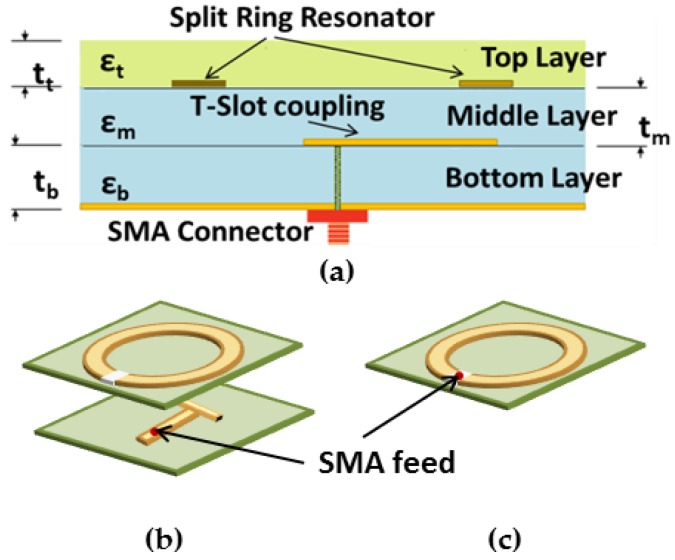
(**a**) Side view of the proposed multilayer sensor. The bottom layer consists of TMM4 substrate and the ground plane, the middle layer consists of TMM4 substrate and the T-patch, and the top layer consists of TMM6 superstrate and the split-ring resonator (SRR) patch; (**b**) Top views of the prototype of the SRR and (**c**) capacitive split-ring resonator (CSRR).

The arrangement of the first sensor consists of three multilayer substrates, as shown in Figure 1. In particular, the sensor consists of a subminiature version A (SMA) connector, coaxially fed T-shaped microstrip line fabricated on the bottom substrate layer, having a dielectric permittivity of 4.5 and a height of 0.64 mm. A split ring is fabricated on the intermediate layer, and the electric field from the ring extends to the tissue through the top superstrate layer, which also acts as a coupling medium. The SRR dimensions include the top layer of superstrate with *W* and *L* = 25.0 mm; the intermediate layer, with *R*1= 8.6 mm and *R*2 = 5.8 mm; and the bottom layer with *T*1= 9.2 mm, *T*2 = 4.2 mm, *W* = 30.0 mm, *L* = 30.0 mm, and the gap width of split-ring resonator, Gap = 0.8 mm. Rogers TMM4 and TMM6 were used for the substrate and superstrate, respectively.

With this done, to go further into sensor optimization, we propose other variants of the ring resonator structure: a Capacitively fed Split Single Circular Ring (CSSCR) resonator. The sensor has two layers—a resonator layer and a matching layer. The resonator layer corresponds to the microstrip resonator structure. The matching layer consists basically of a superstrate which acts as a coupling medium to the target to allow more energy to be radiated into the human tissues and to obtain enhanced and stable resonance characteristics while illuminating the targets. These two sensors were then optimized and employed in clinical measurements.

### 2.2. Clinical Measurement Approach

**Figure 2:**
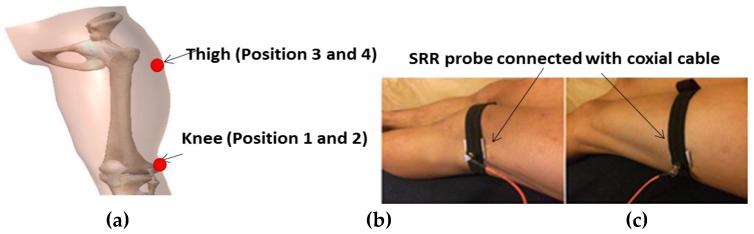
(**a**) The proposed sensor positions. (**b**,**c**) Implemented positions of SRR sensors on the two positions consisting of the distal femur and thigh.

Data were gathered from four consenting volunteers during the Complex Fracture Orthopedic Rehabilitation (COMFORT) measurement campaign at the Telge Rehabilitation Center, Södertälje, Sweden. The data collection was carried out under ethical approval (2016/698-31/1) from Sweden. Since the region around the femur bone is of particular interest in the case of lower extremity injuries, four different positions on the femoral area were used for data collection, as shown in Figure 2a:

1. Anterior of the distal femur 3 cm above the patella.
2. Lateral (outside) of the distal femur.
3. Anterior of the thigh (10 cm above patella) midlength.
4. Lateral of the thigh midlength, same level as the front thigh.

The SRR sensor was attached to each participant’s hip by using a stretchable strap. The sensor was then aligned to the anterior and lateral hip direction as shown in Figure 2b,c and connected to the network analyzer (MiniVNA Tiny-mRS radio solution). The measurement was performed over an operating frequency from 1 to 3 GHz, which is in the range of the operating frequency of the sensor (2.78 GHz) (without tissue surface contact). During clinical measurement, Positions 1 to 4 (distal femur and thigh) were selected for data collection, and further analysis was required to study the penetration depth.

Data collection was done by following the standard protocol at the rehabilitation center. The participants were requested to undergo control measurement using ultrasound (US) with help from a radiologist. The US images we obtained for this clinical measurement were taken from Positions 1 to 4 and are shown in Supplementary Figures S1–S4. The thickness of individual tissues (skin, fat, and muscle) and their composition were obtained from US images of the distal femur and thigh (anterior/lateral) and are summarized in Table 1. 

The anterior and lateral lower extremity was kept immobile during US measurements to reduce artefacts. The data collection was repeated if any change in protocol was detected by the physician. To ensure consistent results, S_11_ was measured three times for any given position. A numerical model was made and validated using the information extracted from US and S-parameter measurements. This model is described in detail in the following subsection. The thickness of each tissue layer was obtained from the US measurement, and can typically be distinguished by observing the grey scale (contrast) between adjacent areas displayed on the US images.

### 2.3 Numerical Model

**Figure 3:**
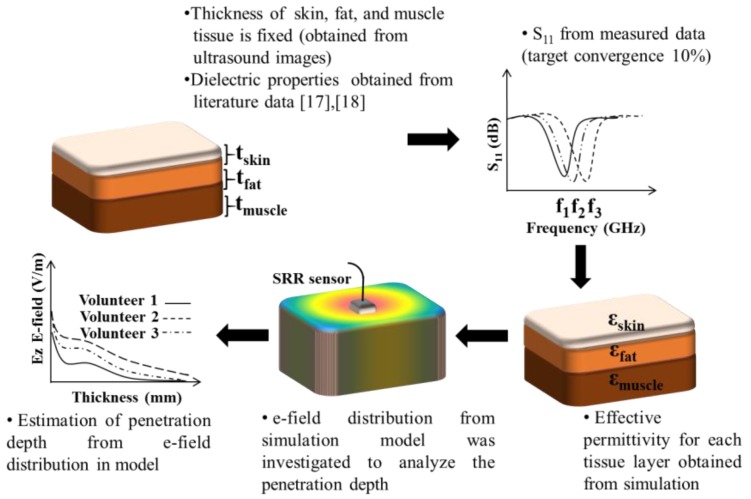
Method of extracting the dielectric profile of different tissues and hence the signal penetration depth using S_11_ data and US images.

The proposed sensor was numerically simulated using Computer Simulation Technology (CST Studio, 2017, SIMULIA, Darmstadt, Germany) based on US tissue thickness measurements. Figure 3 shows the process of extracting the dielectric properties of different tissues and subsequent derivation of signal penetration depth using S_11_ data and US images. The thickness of tissue layers was fixed, the target S_11_ was obtained from measurement, and an initial estimate of dielectric properties was obtained from the literature. Then the dielectric properties were optimized to result in the simulated S_11_ that matches the target measured S_11_ obtained from volunteer measurement with a maximum convergence limit of 10%. The S_11_ data collected from the volunteers were imported into CST in the 1–3 GHz frequency range and were defined as the response of the model. The dielectric parameter of the CST model was optimized for the response thus defined. 

The numerical results have given valuable information on the effective permittivity *ε_eff_* and conductivity σ, expressed in S/m. Specifically, this numerical study demonstrates a characterization of tissue properties over a microwave frequency range 2.4–2.6 GHz. These frequencies overlap with the industrial, scientific, and medical radio (ISM) frequency band, which is free to be used in medical applications. This band was chosen as it covers the frequency range of interest for the sensor and the system. Additionally, this model was developed to estimate the signal penetration depth based on analyzing the E-field distribution in the layered tissue model. In order to characterize the penetration depth in the system, observations were made from the simulated E-field and the results were correlated. The penetration depth offers good information on the stipulated E-field distribution for analyzing sensor performance and at the same time can be used in extensive clinical measurements. 

## 3. Results and Analysis

### 3.1. Permittivity Analysis

The tissue dielectric data reported in literature [22,23,24,25] (for 1–3 GHz) for skin, fat, and muscle were used as initial tissue properties for training (iteration) of the numerical model (as described in Table 2). The real part of relative permittivity, *ε_r_*, and conductivity, *σ*, expressed in S/m were taken into consideration for the dielectric properties of the tissues.

We further investigated the effective permittivity values of tissues by examining each position on a simulated numerical model. In particular, the properties consist of the relative permittivity and conductivity, for which the numerical model was trained. The S_11_ data measured at the different femoral positions of the volunteer were used as the target data to train the proposed model to obtain an effective permittivity for different volunteer tissues. Among all the tissue layers, the muscle layer has the highest dielectric properties and can detune the sensor resonance more. It can be seen that the variation in dielectric properties ∆*ε_r_* and ∆*σ* of Volunteer 1 is 0.2 and 0.06, respectively, compared to the literature data. In particular, for *ε_r_*, the accuracy is within 0.38%, while for *σ*, the accuracy is within 3.45%.

### 3.2. Dependence of Resonance Frequency on Position

**Figure 4:**
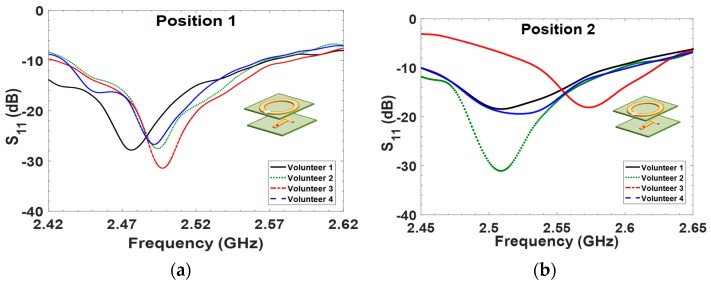

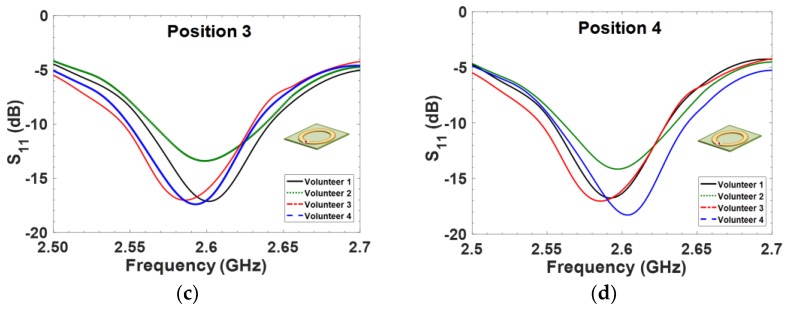
Measured S_11_ data versus frequency response for four volunteers from difference position measurements.

In this section, we present the results of S_11_ data measurements and their analysis. A general trend observed in the experiment is that the tissue thickness and position point influence the resonance frequency of the sensors. In Figure 4a–d, individual measurements from Positions 1 to 4 are presented. For calculations based on the shifted resonance frequency ∆*f_r_*, the highest frequency shift was obtained at Position 1, as shown in Figure 4a, for Volunteer 3 with 30.1 MHz. The lowest calculated ∆*f_r_* is at Position 4 for Volunteer 4 with 17.1 MHz. From these results, it is evident that the variation of tissue thickness at each position has an impact on the resonance frequency shift. 

The influence of material properties can also be observed with respect to resonance frequency, *f_r_*, for various measurement setups. The data shows that the resonance frequency may shift downwards from the free-space resonance frequency of the sensor when thicker muscle tissue is present at the concerned position. The resonance frequency may move towards free-space resonance of the sensor when the muscle layer is thinner. 

Similarly, for Position 3 of Volunteer 3, the ∆*f_r_* was higher, and the corresponding muscle thickness was measured to be 49.7 mm. From all ∆*f_r_* observations, it can be seen that the thicker muscle tissue caused a larger shifting of resonance frequency compared to the thinner muscle tissue.

### 3.3. Penetration Depth Analysis

**Figure 5:**
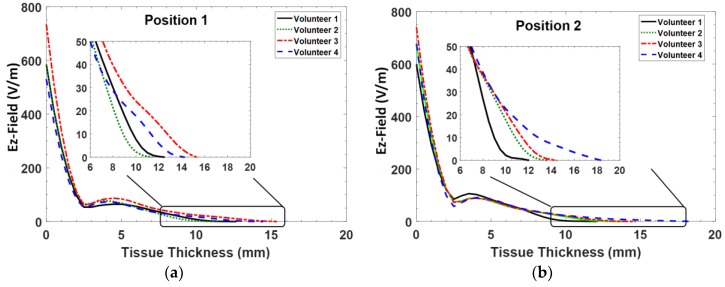

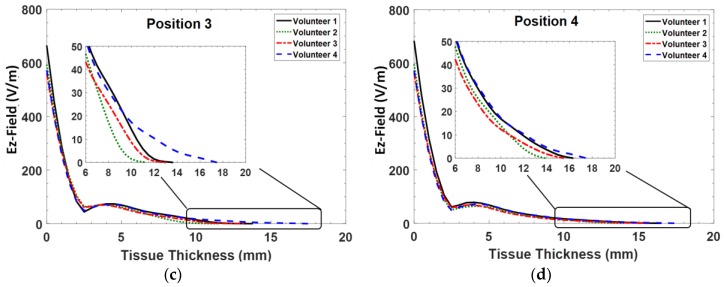
Plot of the E-field penetration as a function of tissue thickness from different positions.

To intuitively analyze and compare the performances of two sensors, the electric field distribution was studied in this section. We further investigated the penetration depth by examining each position of items of interest. Figure 5a,b show the results of the E-field distribution with respect to tissue thickness. The variation in the E-field distribution across different tissues for Positions 1 and 2 shows the penetration depth of the SRR sensor for the respective positions. In this section, the actual position of E-field was obtained from the *Ez* axis. The E-field along the *Ez* axis perpendicular to the sensor plane was considered. The starting point of the *Ez* axis was taken at the maximum E-field strength which happens to be at the interface of the sensor and skin surface. The distribution of the E-field was observed along the *Ez* axis through the different tissue layers of the volunteer until the E-field died off. In this case, the penetration depth was obtained from 11.5 to 18 mm. According to the data from tissue thickness based on US measurements, the total E-field covered at least 46.3% of the distance from the skin to the muscle boundary.

The differences in penetration depth at Positions 3 and 4 are plotted in Figure 5c,d. As shown in Figure 5d, the changing trend of penetration depth was similar between all the volunteer measurements. Considering the data of tissue thickness, the penetration depth was found to be higher when a thicker fat layer is present. Thus, it is observed that the thickness of the fat layer had a great impact on the E-field distribution. Since fat tissue has inherently low water content it shows very low frequency dispersion in its dielectric properties. It can be seen in Figure 5a–d that the E-field extends throughout the fat layer (2.5–10 mm on average on the tissue thickness axis) and the E-field intensity increases at the boundary between skin and fat. Therefore, the thickness of the fat tissue has a higher significance on the measured S_11_ values compared to its dielectric properties. In this scenario, at the operating frequency of 2.45 GHz, Volunteer 4 gives a higher indication of a larger penetration depth, calculated to be 18.5 mm. As mentioned, the penetration depth demonstrates a clear dependence with tissue thickness, especially for the fat layer. The depth was gradually decreased once the E-field arrives at an average fat thickness of 9.2 mm. This is due to the fact that E-field penetrates more in the fat tissues because of its lower conductivity. 

On the other hand, considering the fact that the tissue properties are those of a lossy medium [22,23,24], the E-fields are induced in the human body when the human body is exposed to the RF electromagnetic field [26,27,28]. Therefore, as a result, the signal penetration depth for this measurement scenario depends on the thickness of tissue with lower conductivity such as the fat layer. The magnitude of the E-field that enters the fat tissue will not attenuate quickly. The thickness of fat tissue does not impact the proportion of the field that is transmitted into it; only the contrast between the skin and the fat and the thickness of the first layer the field has travelled through (skin) determine the field magnitude that propagates into the fat layer. 

Furthermore, the correlation between E-field and distance can be explained by using the relationship of power propagation and its absorption in the fat layer. As the thickness is increased, the power will be more easily transmitted into fat because of its lower loss compared to other tissue layers. Therefore, the E-field extent represents the percentage increase in the E-field magnitude in the fat layer. It is calculated using the following equation:

(1) E−Field Extent=((MaxMin)−1)× 100%

where Max is the maximum E-field in the fat layer and Min is the minimum E-field at the skin–fat interface. 

**Figure 6:**
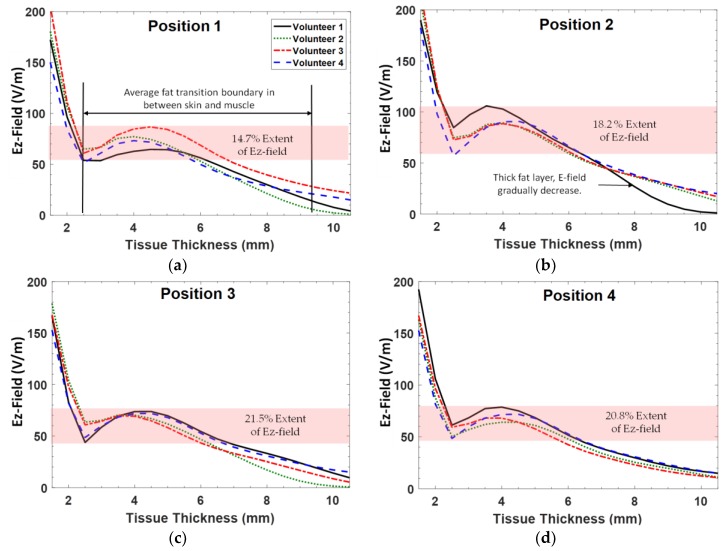
E-field penetration from fat transition boundary in between skin and muscle.

Moreover, there was lower E-field propagation at Positions 1 and 2 as shown in Figure 6a,b, respectively. Thus, the E-field propagation at Position 2 for Volunteer 1 decreased rapidly due to a small transition in between the fat and muscle boundary.

On the other hand, as shown in Figure 6c,d, the E-field propagation at Positions 3 and 4 was almost the same in the range of a 20.8–21.5% increase in attenuation level. All these scenarios might be explained by [29,30], which is related to the influence of the fat layer as a channel medium for intrabody communication. According to these findings, the signal transmission can be improved with the thickness of the fat and muscle tissues. The signal transmission is analyzed with respect to the length and thickness of the tissue channel.

From one side it is clear that the CSRR sensor shows better penetration characteristics since there is more electric field energy penetrating the different tissue layers, showing an improvement with respect to its predecessor, the SRR structure. From the other side it is possible to see that SRR has a better field distribution in muscle and bone, making it preferable for distal femur positions, while CSRR is preferable for thigh positions because of its higher energy in the fat layer.

## 4. Conclusions

In this paper, with the participation of four consenting volunteers, S_11_ measurements were made and tissue thickness information from US images was obtained for the femoral area. Numerical models were created and the effective permittivity of individual tissues was derived. The numerical model was used to simulate the E-field distribution following a penetration depth calculation. It was observed that the resonance frequency has substantial dependence on the specific measurement position on the femoral area. The penetration depth at any given position also depends on the thickness of tissue with lower conductivity, such as the fat layer. As a key result of this study, this proposed method can be used to estimate penetration depth by using sensor S_11_ measurements and tissue thickness provided from US images. Preliminary results are, therefore, encouraging and suggest the effectiveness of using noninvasive methods to monitor tissue variations, especially during lower extremity fracture rehabilitation.

## Figures and Tables

**Table 1:** Ultrasound (US) measurement of different tissue thicknesses from volunteers.

Volunteer	Layer	Position 1 (mm)	Position 2 (mm)	Position 3 (mm)	Position 4 (mm)
1	Skin	3.0	1.9	2.6	2.3
	Fat	6.1	5.8	7.2	11.5
	Muscle	15.4	14.4	35.5	28.6
2	Skin	2.3	2.3	2.4	2.7
	Fat	5.6	8.0	5.3	8.7
	Muscle	16.3	18.0	44.6	37.2
3	Skin	2.9	2.4	2.3	2.3
	Fat	10.1	8.8	7.2	11.0
	Muscle	19.7	23.5	49.7	44.2
4	Skin	2.5	2.5	2.5	2.5
	Fat	8.9	14.3	20.1	20.4
	Muscle	11.7	16.7	35.6	24.2

**Table 2:** Numerical data for different effective permittivity and conductivity.

Volunteer	Layer	Literature [25]		Positions 1 and 2 (derived dielectric properties)		Positions 3 and 4 (derived dielectric properties)	
		*ε_r_*	*σ* (S/m)	*ε_r_*	*σ* (S/m)	*ε_r_*	*σ* (S/m)
/	Skin	38.0	1.5	/			
	Fat	5.3	0.1				
	Muscle	52.7	1.74				
1	Skin	/		34.2	1.3	38.0	1.5
	Fat			5.3	0.1	5.3	0.1
	Muscle			58.0	1.9	52.9	1.8
2	Skin	/		37.9	1.4	31.9	1.2
	Fat			5.2	0.1	6.2	0.1
	Muscle			52.9	1.7	61.2	2.1
3	Skin	/		34.2	1.3	30.4	1.2
	Fat			4.8	0.11	4.7	0.12
	Muscle			58.0	1.9	52.9	1.8
4	Skin	/		34.1	1.2	30.2	1.3
	Fat			4.7	0.12	5.27	0.15
	Muscle			57.9	1.9	52.6	1.75

## Supplementary Materials

The following are available online at http://www.mdpi.com/1424-8220/18/2/636/s1, Figure S1: Measured tissue thickness of (a) position 1 (b) position 2 (c) position 3 and (d) position 4 for Volunteer 1 performed using Ultrasound tool. Figure S2: Measured tissue thickness of (a) position 1 (b) position 2 (c) position 3 and (d) position 4 for Volunteer 2 performed using Ultrasound tool. Figure S3: Measured tissue thickness of (a) position 1 (b) position 2 (c) position 3 and (d) position 4 for Volunteer 3 performed using Ultrasound tool. Figure S4: Measured tissue thickness of (a) position 1 (b) position 2 (c) position 3 and (d) position 4 for Volunteer 4 performed using Ultrasound tool.
