# Supplementary material for: Split-Ring Resonator Sensor Penetration Depth Assessment Using In Vivo Microwave Reflectivity and Ultrasound Measurements for Lower Extremity Trauma Rehabilitation
Source: Sensors (Basel). 2018 Feb 21;18(2):636. doi: 10.3390/s18020636 (PMC5855979; doi:10.3390/s18020636)
Supplement: Supplementary file 1 [file sensors-18-00636-s001.pdf]

Supplementary Note 1

In this section we describe in detail each US image obtained from four different volunteers.

Ultrasound (US) image descriptions: Supplementary Figures S1–S4 show the different position from each image as well as the tissue thickness for skin, fat, and muscle. The very different characteristics of the tissue layers and their appearance are noticeable, but this will be even more obvious as we later move on to study the dielectric properties and penetration depth of the E-field in the main article.

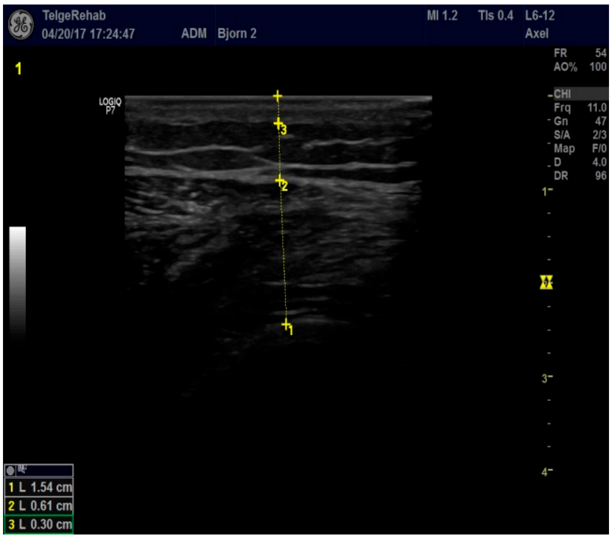

(a)

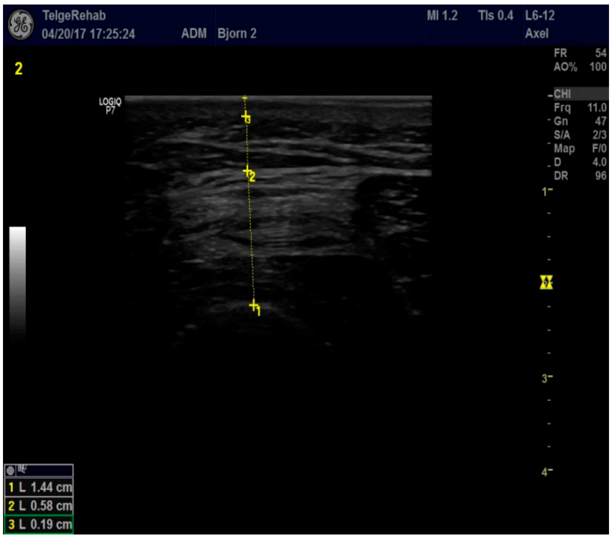

(b)

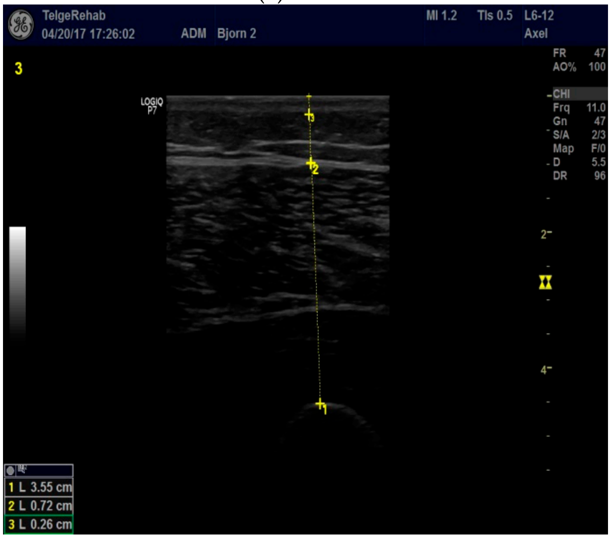

(c)

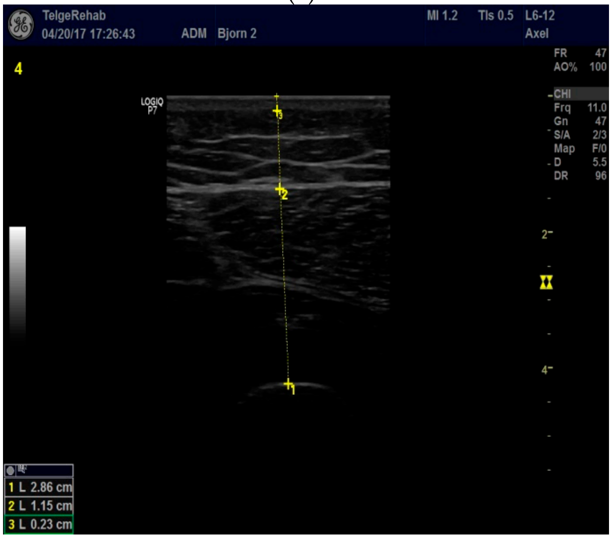

(d)

Figure S1. Measured tissue thickness of (**a**) Position 1, (**b**) Position 2, (**c**) Position 3, and (**d**) Position 4 for Volunteer 1 performed using the Ultrasound tool.

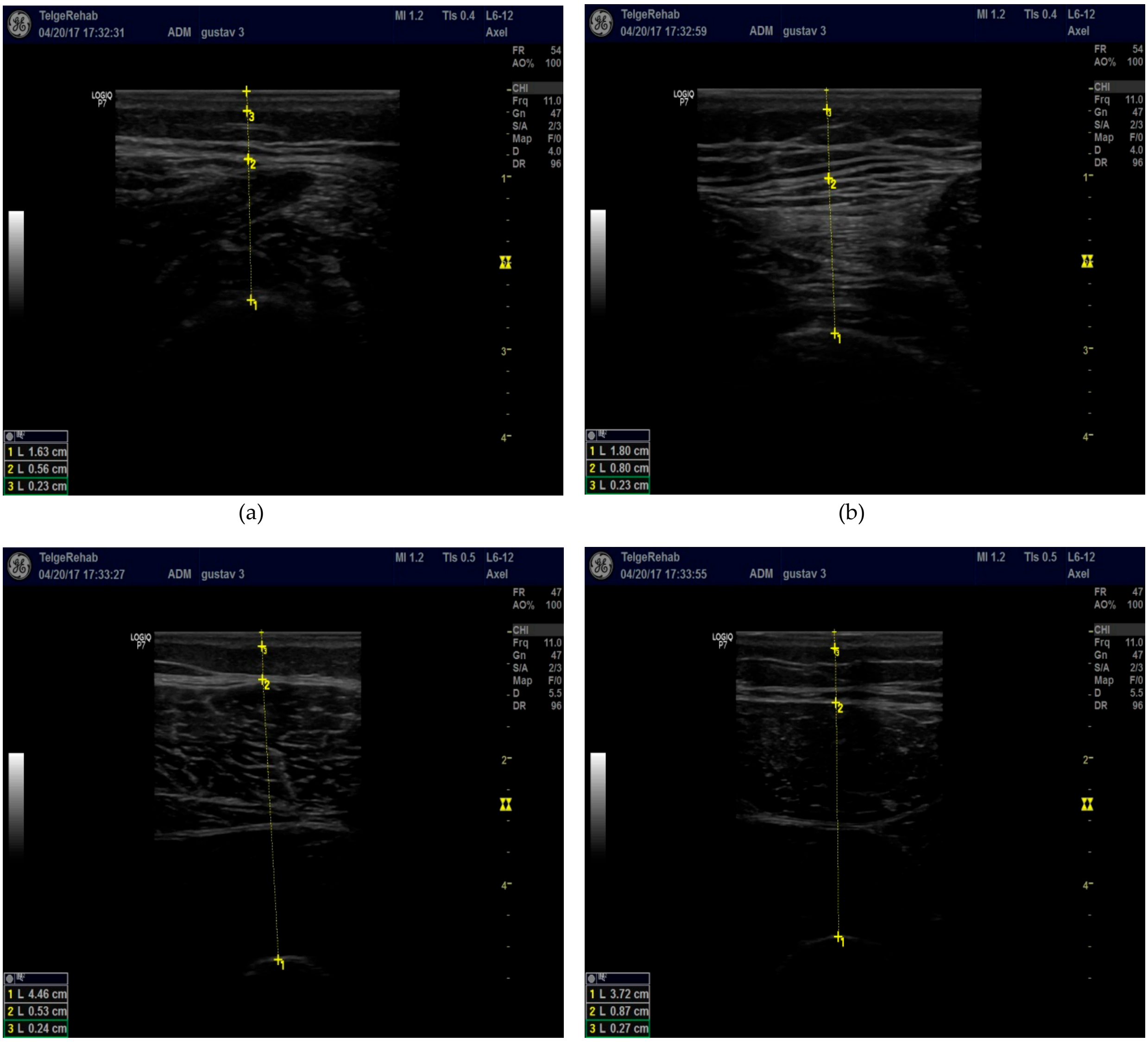

(a) (b) (c) (d)

Figure S2. Measured tissue thickness of (**a**) Position 1, (**b**) Position 2, (**c**) Position 3, and (**d**) Position 4 for Volunteer 2 performed using the Ultrasound tool.

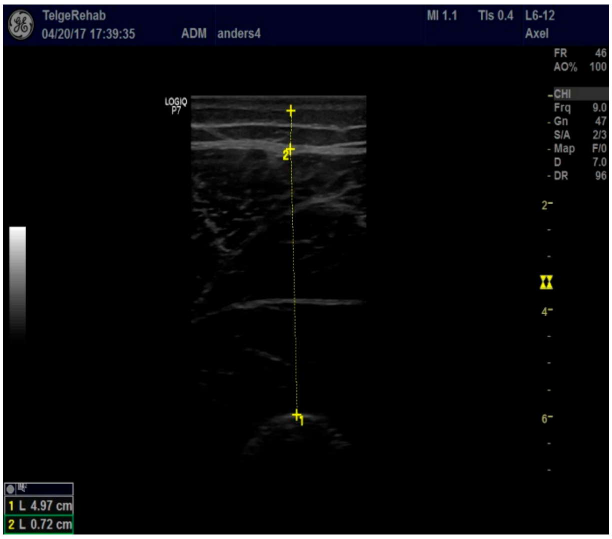

(a)

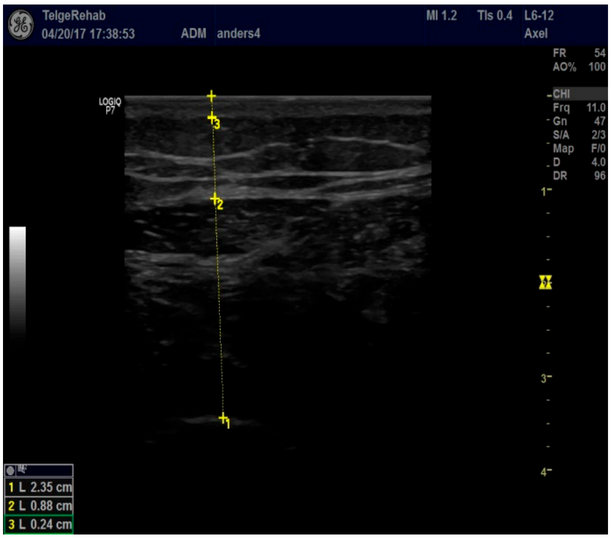

(b)

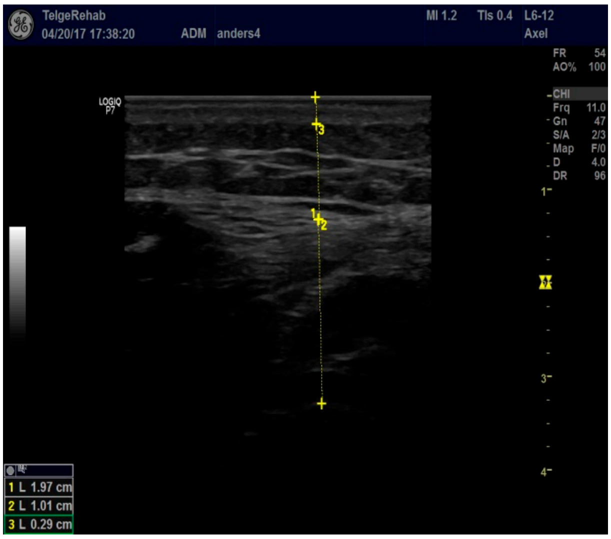

(c)

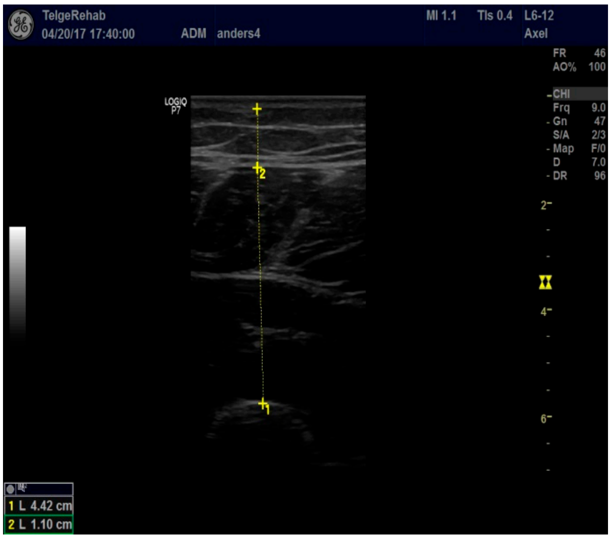

(d)

Figure S3. Measured tissue thickness of (**a**) Position 1, (**b**) Position 2, (**c**) Position 3, and (**d**) Position 4 for Volunteer 3 performed using the Ultrasound tool.

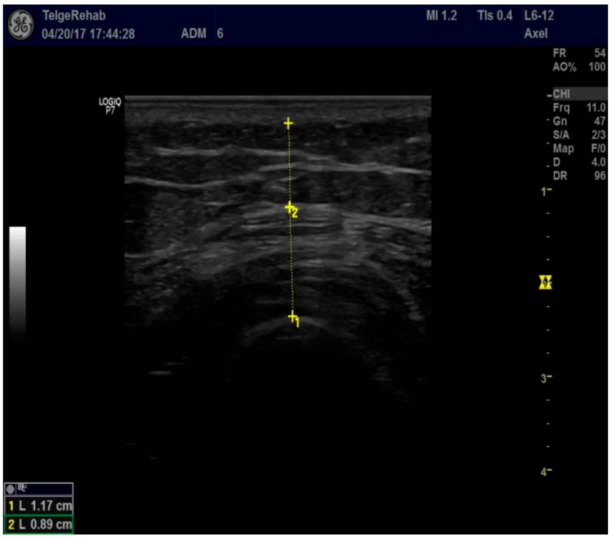

(a)

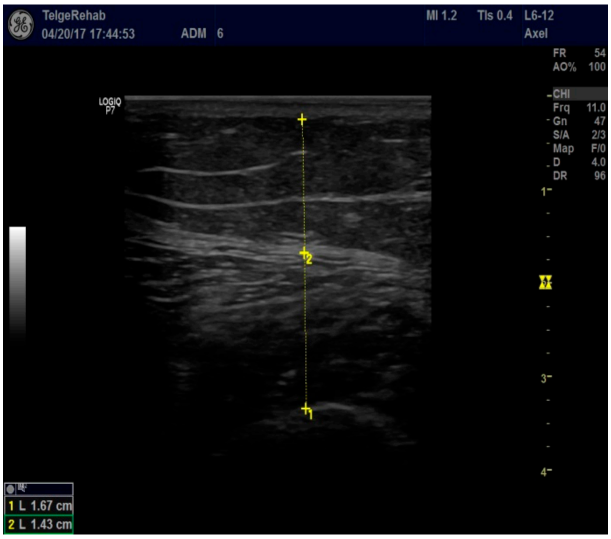

(b)

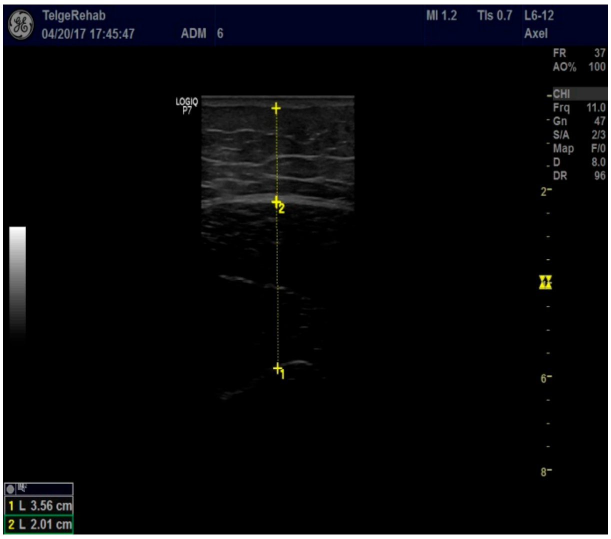

(c)

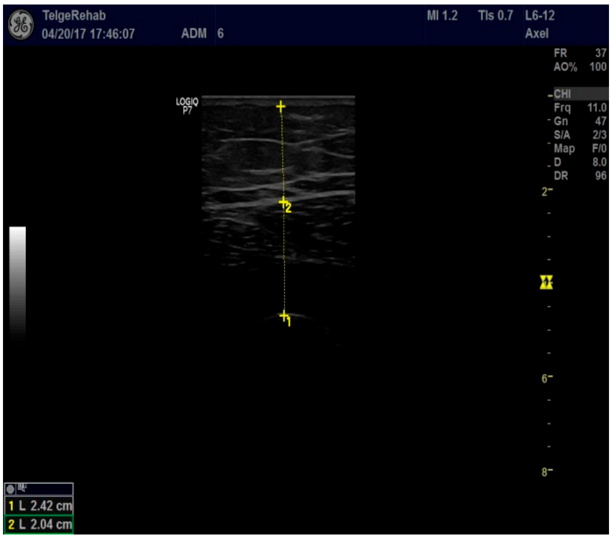

(d)

Figure S4. Measured tissue thickness of (**a**) Position 1, (**b**) Position 2, (**c**) Position 3, and (**d**) Position 4 for Volunteer 4 performed using the Ultrasound tool.
